# Characterization of Etiology and Pattern of Dental Extraction among Patients Presenting to Dental Department of a Tertiary Care Centre: A Descriptive Cross-sectional Study

**DOI:** 10.31729/jnma.8745

**Published:** 2024-09-30

**Authors:** Mamata Shakya, Bhoj Raj Adhikari, Nitesh Chaurasia, Chandan Upadhyaya, Dilip Prasad Rauniyar, Arjun Hari Rijal, Manisha Nepal

**Affiliations:** 1 Department of Oral and Maxillofacial Surgery, Kathmandu University School of Medical Sciences, Dhulikhel, Kavre, Nepal; 2 Department of Oral Medicine and Maxillofacial Pathology, Kathmandu University School of Medical Sciences, Dhulikhel, Kavre, Nepal; 3 Department of Periodontology, Kathmandu University School of Medical Sciences, Dhulikhel, Kavre, Nepal; 4 Department of Conservative Dentistry and Endodontics, Kathmandu University School of Medical Sciences, Dhulikhel, Kavre, Nepal

**Keywords:** *endodontics*, *Nepalese*, *periodontitis*, *tooth extraction*

## Abstract

**Introduction:**

Dental extraction is a common curative treatment against pain provided to the patients. A limited number of scientific literature are available that characterizes the reasons for teeth extractions in Nepalese population. The aim of this study is to find out the causes for dental extraction in patients and explore the relationships between demographic variables and loss of teeth in the Nepalese population.

**Methods:**

This is a descriptive cross-sectional study performed to assess the predictors and reasons for permanent tooth extraction among the Nepalese patients who visited dental department in a tertiary health care center from 18^th^ June 2022 to 17^th^ June 2023. Ethical approval was obtained from the Institutional Review Committee (Approval No: 52/22). Demographic details were recorded, diagnosis established and the teeth in question were extracted. Descriptive statistical analysis was performed.

**Results:**

A total of 465 (42.66%) permanent teeth were extracted from the 1090 patients, out of which 198 (18.16%) were male and 267 (24.49%) were female patients between the age of 10 years to 70 years. Among all the participants, 165 (15.13%) extraction were done due to peridontal disease and 213 (19.54%) of extraction was done due to endodontic disease.

**Conclusions:**

Endodontic and periodontal diseases were the most common causes of dental extraction. Permanent molars and incisors were extracted the most.

## INTRODUCTION

Globally, three and a half million people suffer from oral health diseases, mainly untreated dental caries, severe periodontal diseases, loss of teeth and oral cancer, all leading to edentulism.^1^ Edentulism can lead to masticatory dysfunction, pronunciation difficulty, malnutrition, limited choices of food, social isolation, and poor quality of life.^2^ Loss of teeth is a good indicator of degree of oral hygiene.^3-5^

Infection, pain and discomfort can have a significant influence on general well-being and therefore poor oral health has a detrimental influence on the quality of life.^6-10^ Dental extraction is a common curative treatment against pain provided to the patients.^11^ A limited number of scientific literature are available that characterizes the reasons for teeth extractions in Nepalese population.^12,13^

The aim of this study is to find out the causes for dental extraction in patients visiting Kathmandu University School of Medical Sciences, Dhulikhel Hospital (KUSMS-DH).

## METHODS

This is a descriptive cross-sectional study performed to assess the predictors and reasons for permanent tooth extraction among the Nepalese patients who visited the dental department in KUSMS-DH, Dhulikhel, Kavre from 18^th^ June 2022 to 17^th^ June 2023. Ethical approval was obtained from the Institutional Review Committee of the same institute (IRC-KUSMS Approval No: 52/22) and informed consent was taken from all the study participants.

All the patient visiting dental department in KUSMS-DH during the study period, providing informed consent were included in this study. Primary teeth (if present) and partially erupted teeth were excluded from the present study. After obtaining informed and written consents from the participants, name, gender, age, address, and educational level were recorded. At the time of examination, medical and dental histories of the participants were reviewed. Dental history including the history and causes of permanent tooth extraction were noted down. Periodontal and dental examinations were performed on permanent teeth only. The gingival, periodontal and periapical examination were performed, that consisted of measurement of the gingival and periodontal supporting tissue, including gingivitis, attachment loss, and probing pocket depth (using the Williams probe), percussion test, and periapical radiograph (where necessary). An assessment of tooth mobility, gingival bleeding, dental plaque, dental calculus, gingival recession, draining sinus, swelling and discoloration of teeth were also performed. The teeth in question were extracted under local anesthesia using standard operative procedure under aseptic condition.

All the available information were entered in Microsoft Excel and analyzed using IBM SPSS. The obtained data were analyzed with major focus on demographic details, education level, pattern and causes of dental extraction. Descriptive statistical analysis was performed.

## RESULTS

A total of 1090 patient visited the dental department during this study period. Among them 465 (42.66%) patients had undergone extraction of permanent teeth, out of which 198 (18.16%) were from male and 267 (24.49%) were female. The age ranged from 10 years to 70 years old individuals with a mean age of 47±1.51 (Table 1).

**Table 1:** Demographic detail of the patients visiting dental department of KUSMS-DH (n = 1090).

Demographic details	n (%)
**Gender**	
Male	472 (43.30)
Female	618 (56.70)
**Mean Age**	471.51 years

(KUSMS-DH) Kathmandu University School of Medical Sciences, Dhulikhel Hospital

History, clinical examination and diagnosis of the individual tooth suggested that in 165 (35.48%) patients, tooth extraction was done because of periodontal diseases, while in 213 (45.81%) it was done for endodontic diseases, and in the remaining 87 (18.71%) patients tooth extraction was done for other reasons including traumatic injury, pericoronitis, orthodontic and pre-prosthodontic treatment planning. Tooth extraction due to periodontal problem was 69 (14.84%) in the age group 51 to 60 years, while 63 (13.55%) of extractopm were due to endodontic reason in the age group of 31 to 50 years (Table 2).

**Table 2:** Age distribution ofmajor causes ofteeth extraction calculated from the patient whounderwent extraction (n=465).

Age Distribution (years)	Causes of teeth extraction			No of teeth extracted n(%)
	Periodontal Diseases n(%)	Endodontic Diseases n(%)	Others n(%)	
10 to 20	0 (0.00)	8 (1.72)	1 (0.22)	9 (1.94)
21 to 30	2 (0.43)	21 (4.51)	11 (2.36)	34 (7.30)
31 to 40	8 (1.72)	63 (13.55)	10 (2.15)	81 (17.42)
41 to 50	45 (9.67)	46 (9.90)	15 (3.23)	106 (22.80)
51 to 60	69 (14.84)	32 (6.88)	29 (6.24)	130 (27.96)
61 to 70	41 (8.82)	43 (9.25)	21 (4.51)	105 (22.58)
Total	165 (35.48)	213 (45.81)	87 (18.71)	465 (100)

Among the teeth that were extracted, 164 (35.27%) were molars and 148 (31.83%) were incisors (Table 3).

**Table 3:** Pattern of teeth extraction calculated from the patient who underwent extraction (n = 465).

Age distribution (years)	Extracted tooth			
	Incisors n (%)	Canines n (%)	Premolars n (%)	Molars n (%)
10 to 20	1 (0.22)	-	-	8 (1.72)
21 to 30	2 (0.43)	-	12 (2.58)	20 (4.3)
31 to 40	12 (2.58)	-	10 (2.15)	59 (12.69)
41 to 50	40 (8.60)	8 (1.72)	27 (5.80)	31 (6.67)
51 to 60	50 (10.75)	25 (5.37)	23 (4.95)	32 (6.88)
61 to 70	43 (9.25)	35 (7.53)	13 (2.80)	14 (3.01)
Total	148 (31.83)	68 (14.62)	85 (18.28)	164 (35.27)

There were 132 (28.39%) extractions in female and 81 (17.42%) in male due to endodontic disease (Table 4).

**Table 4:** Causes of teeth extraction depending upon the gender calculated from the patient who underwent extraction (n = 465).

Gender	Cause of Teeth extraction			Total; n(%)
	Periodontal disease n (%)	Endodontic disease n (%)	Others n (%)	
Male	78 (16.77)	81 (17.42) 39 (8.39)	198 (42.58)
Female	87 (18.71)	132 (28.39)	48 (10.32)	267 (57.42)
Total; n(%)	165 (35.48)	213 (45.81)	87 (18.71)	465 (100)

## DISCUSSION

The results of the current study showed that endodontic and periodontal diseases are the foremost reasons for tooth extractions at KUSMS-DH. This finding is in agreement with the majority of previous studies conducted within the similar geographical region,^14-16^ as well as internationally,^5,17-19^ where endodontic and periodontal diseases and their sequels were the primary reasons for teeth extraction.

The number of tooth extraction in the present study was higher among older population. More than half of the teeth extraction was observed in the age group of 51 to 70 years old which is similar to other studies.^5,19^ Remarkably, earlier studies have shown that the tooth loss due to periodontal disease was more common in this age group as compared to that in younger populations.^16^ This finding has been attributed to multiple age-related factors including the presence of systemic conditions and medications for the same, problem with maintaining oral hygiene secondary to diminishing manual dexterity and genetic proneness to periodontal diseases.^20^

Incisors and premolars were extracted mainly during 41 to 60 years of age. Molars were lost in greater number during 31 to 40 years of the age of the patients. Molar teeth had a high rate of dental caries mainly attributed to their morphological features including presence of deep pits and fissures on their occlusal surface. Permanent first molars are the first permanent teeth to erupt in the oral cavity. They erupt at the age of 6-7 years and are hence are neglected by the parents and the children. This might be another important reason for high rate of dental caries in molars. Anterior teeth including incisors and canines are less prone to dental caries because of their self-cleansing property attributed to their morphological features and location in the oral cavity.^18^ Deep dental caries, loss of tooth structure, and periapical infection as sequels of dental caries were the main reason that the molars were extracted. As a result, we found high rate of endodontic diseases as a major cause of teeth loss in the present study. Additionally, children having dental caries in their primary teeth were three times more likely to develop caries in their permanent teeth.^21^ This fact in present study population is further supported by our earlier studies where we found high prevalence of dental caries in the deciduous molars in children visiting to the same center for treatment.^22^

Endodontic causes, thereby, was the main cause of tooth extraction in patients aged 31 to 40 years. Our result is consistent with the findings presented by previous researchers.^16^ Several previous studies have shown that irregular dental visits, failing to comply with oral hygiene instructions, and high sugar intake are the main attributing factors for the dental caries and associated endodontic diseases in early adulthoods.^6,23^ Meta-analysis and systemic review of studies in Indian populations showed an overall prevalence of dental caries in individuals under 18 years of age to be 52%, thereby supporting our study.^24^ In the current study, total female population (56.70%) were more than male population with the similar distribution (57.42%) in the pool of patients who underwent extraction. Amongst the female who underwent extraction, endodontic disease were more (28.39%). It is not possible to mention that female patients displayed higher chances of endodontic diseases, with this findigs. However, studies have rationalized the higher number of tooth extraction among females due to less interest in adhering to dental maintenance, failure to recall treatment appointments, getting busy in household activities, economical reason, lack of transportation facilities and personal or health related issues.^25-27^

Likewise, out of total population visiting the hospital, most (66.60%) of the patient were educated with similar distribution (60%) in the pool of patients who underwent extraction. Amongst the educated patients who underwent extraction, endodontic disease were more (29.68%%). Our study has several limitations. The study is not designed and sample size has not been calculated to prove the association, therefore statistical test for association has not been done. This study does not prove the relationship of gender and educational status with the cause of dental extraction. This can be the future area of study. Moreover, this study has taken the total sample over a period of time, therefore the findings is applicable to the given period and cannot be generalised beyond this time period.

## CONCLUSION

Endodontic and periodontal disease are found to be the main causes of permanent teeth extraction in Nepalese patients visiting KUSMS-DH. Molars and Incisors were the most commonly extracted teeth.

## References

[1] Jain N, Dutt U, Radenkov I, Jain S (2024). WHO's global oral health status report 2022: Actions, discussion and implementation.. Oral Diseases..

[2] Gbolahan OO, Fasola AO, Aladelusi TO (2019). Attitude and Behavior to Oral Health of 456 Patients Who Presented for Tooth Extraction at 2 Health Facilities in Southwestern Nigeria.. J Patient Exp..

[3] Kawachi I, Kennedy BP (1999). Income inequality and health: pathways and mechanisms.. Health Serv Res..

[4] Neto JMS, Nadanovsky P (2007). Social inequality in tooth extraction in a Brazilian insured working population.. Community Dent Oral Epidemiol..

[5] Chrysanthakopoulos NA (2011). Reasons for extraction of permanent teeth in Greece: a five-year follow-up study.. Int Dent J..

[6] Khanal S, Shah P, Ghimire P (2021). Burden of Dental Caries in the Nepalese Children: A Literature Review.. J Nepal Assoc Pediatr Dent..

[7] Shekhawat KS, Chauhan A, Nordstroem M (2016). Dental pain and its impact on quality of life among indigenous adolescents of Himalayas (Ladakh), India.. Indian J Dent Res..

[8] Yang SE, Park YG, Han K, Min JA, Kim SY (2015). Dental pain related to quality of life and mental health in South Korean adults.. Psychology, health & medicine..

[9] Paulo S.A., Goes RH, Richard G Watt, Sheiham A (2008). Impacts of dental pain on daily activities of adolescents aged 14-15 years and their families.. Acta Odontologica Scandinavica..

[10] Hugo FN, Hilgert JB, Da Luz Rosario de Sousa M, Cury JA (2009). Oral status and its association with general quality of life in older independent-living south-Brazilians.. Community Dent Oral Epidemiol..

[11] Khapung A, Rao G N, Shrestha S (2020). Reasons and patterns of permanent teeth loss among patients attending a dental college in Kathmandu.. J Nepal Soc Perio Oral Implantol..

[12] Mahat AK, Basnet LB, Puri R, Shrewastwa MK, Panta N (2023). Predictors of Preventable Tooth Loss due to Extraction-A Cross-Sectional Study.. Nepal Med J..

[13] Cunha AR da, Bastos LF, Iser BPM, Malta DC, Goes PSA de, Hugo FN (2022). Toothache and tooth extraction as reasons for dental visits: an analysis of the 2019 National Health Survey.. Braz Oral Res..

[14] Haseeb M, Ali K, Munir MF (2012). Causes of tooth extraction at a tertiary care centre in Pakistan.. J Pak Med Assoc..

[15] Shakeel J, Siddiq M, Mushtaq O, Raza SA, Rauf Z, Rathi AK (2022). Pattern of tooth extraction in a Tertiary Care Hospital-Karachi, Pakistan.. Professional Med J..

[16] Shah A, Faldu M, Chowdhury S (2019). Reasons for extractions of permanent teeth in western India: A prospective study.. Int J Appl Dent Sci..

[17] Passarelli PC, Pagnoni S, Piccirillo GB, Desantis V, Benegiamo M, Liguori A (2020). Reasons for Tooth Extractions and Related Risk Factors in Adult Patients: A Cohort Study.. Int J Environ Res Public Health..

[18] Jafarian M, Etebarian A (2013). Reasons for extraction of permanent teeth in general dental practices in Tehran, Iran.. Med Princ Pract..

[19] Al-Shammari KF, Al-Ansari JM, Al-Melh MA, Al-Khabbaz AK (2006). Reasons for tooth extraction in Kuwait.. Med Princ Pract..

[20] Chatzopoulos GS, Jiang Z, Marka N, Wolff LF (2024). Periodontal Disease, Tooth Loss, and Systemic Conditions: An Exploratory Study.. Int Dent J..

[21] Li Y, Wang W (2002). Predicting caries in permanent teeth from caries in primary teeth: an eight-year cohort study.. J Dent Res..

[22] Adhikari BR, Shakya M, Bhatta N, Upadhya S, Mahanta SK (2023). Dental Caries on Deciduous Molars among Children Visiting Dental Outpatient Department of a Tertiary Care Centre.. J Nepal Med Assoc..

[23] Alhabdan YA, Albeshr AG, Yenugadhati N, Jradi H (2018). Prevalence of dental caries and associated factors among primary school children: a population-based cross-sectional study in Riyadh, Saudi Arabia.. Environ Health Prev Med..

[24] Pandey P, Nandkeoliar T, Tikku AP, Singh D, Singh MK (2021). Prevalence of Dental Caries in the Indian Population: A Systematic Review and Meta-analysis.. J Int Soc Prevent Communit Dent..

[25] Skaret E, Raadal M, Kvale G, Berg E (2000). Factors related to missed and cancelled dental appointments among adolescents in Norway.. Eur J Oral Sci..

[26] Shabbir A, Alzahrani M, Khalid AA (2018). Why do patients miss dental appointments in eastern province military hospitals, Kingdom of Saudi Arabia?. Cureus..

[27] Anagha KA, Megha M, Karuveettil V, Kumar SV (2024). Perceptions of barriers towards dental appointment keeping among patients of a tertiary care setting: A mixed method exploration.. J Oral Biol Craniofac Res..

